# Exploring Autistic People’s Experiences of and Attitudes Towards Cervical Screening: A Mixed-Methods Study

**DOI:** 10.1177/13623613261439937

**Published:** 2026-04-24

**Authors:** Shona Murdoch, Bethany Donaghy, Aimee Grant, Kayleigh Sheen, David John Moore

**Affiliations:** 1 Liverpool John Moores University, UK; 2 Swansea University, UK; 3 The University of the West of England, UK

**Keywords:** autism, cancer screening, cervical screening, mixed methods, theory of planned behaviour

## Abstract

Cervical screening can be lifesaving, yet attendance rates are lower than recommended within the general population and even lower within the autistic population. There is currently no published research systematically exploring autistic people’s cervical screening experiences. This research aimed to explore the experience of cervical (“smear”) screening for autistic people in the United Kingdom. Autistic people (*N* = 97) completed an online mixed-methods questionnaire about their cervical screening experiences. Questions considered experiences of pain, sensory and communication issues, knowledge of cervical cancer, attitudes towards screening, and experience of sexual assault. Findings suggest that an autistic person’s intention to attend their screening is important to understand their actual attendance at the screening. Quantitatively, pain, sensory and communication issues, or knowledge of cervical cancer were not associated with screening attendance. However, qualitatively, they were. Two themes emerged: “Communication disconnect across the care journey” and” Echoes of the past: the lasting impact of previous care encounters” were discussed as barriers to screening engagement. This research highlights the need to improve healthcare communication and other accessibility needs for autistic people when attending cervical screening and for further development of appropriate measurement tools. More research is needed to further inform methods of improving cervical screening services for autistic people.

**Lay Abstract:**

Cervical screening (“smear tests”) can prevent the development of cervical cancer by spotting the signs early. These screening tests can be lifesaving. A large number of the general population do not attend their cervical screening test when invited, and this is even higher for autistic people. One problem is that there is no research to understand why autistic people might not attend their smear tests. We asked autistic people in the United Kingdom to complete a questionnaire online to see who has attended their smear test when invited and looked at different things that might be important in this decision. Pain, sensory issues, and knowledge of cervical cancer did not seem to be important in explaining who did and did not attend a screening appointment. Communication (before, during, and after the screening tests) and previous negative experiences of healthcare (both in general and previous cervical screening tests) were important. This research further highlights the need for more training for healthcare providers in communication for diverse communities and communication needs. More research is also needed to better understand autistic people’s cervical screening and wider healthcare experiences.

## Introduction

Autistic people experience complex health needs and greater mortality rates (Huang et al., 2020) in comparison to neurotypical people. Despite this, autistic people have a greater number of unmet health needs and receive less preventive healthcare (Nicolaidis et al., 2013). A disparity of appropriate healthcare for autistic people is partially explained by reports of increased anxiety upon contact with healthcare services, communication difficulties, and self-reported experiences of perceived stigma (Crane et al., 2019; Lum et al., 2014). A further challenge includes limited knowledge of autism among healthcare professionals (HCPs; Corden et al., 2021), which can lead to a reduced confidence in managing co-occurring needs (Morris et al., 2019). It is plausible that this may hold further implications for adult attendance at preventive check-ups and routine screening care, as has been previously identified with children (Boshoff et al., 2021).

Approximately nine new cervical cancer cases are diagnosed every day in the United Kingdom (Cancer Research UK, 2015). National preventive and screening programmes are undertaken in the United Kingdom, comprising the human papillomavirus vaccination (HPV) vaccination programme for school-aged females and regular cervical screening programmes for females aged over 25 years (with invitations distributed from 24 years). Concerningly, uptake for cervical screening in England has declined annually and failed to reach the 80% uptake target since 2005 (Douglas et al., 2016). Reasons for non-attendance at cervical screening in a general population include perceived pain, embarrassment, and difficulty finding time to attend an appointment (Wilding et al., 2020). The quality of interaction with healthcare providers has also been implicated (Chorley et al., 2017). A negative experience also holds implications for attendance at future appointments (Marlow et al., 2018).

Cervical screening rates are significantly lower in autistic adults compared to non-autistic peers in Sweden (Hu et al., 2023) and South Korea (Shin et al., 2018), and only 38% of an eligible American sample reported undergoing cervical screening (Chen et al., 2024). Limited research has focused on barriers to accessing, and the experiences of, cervical screenings in neurodivergent people and those with developmental disabilities and/or intellectual disabilities (IDs). For clarity, although some autistic people may also have an ID, it is important to make clear that autism itself is not an ID; however, the relevance of such barriers may remain. A recent systematic review (*N* = 16 studies) examining barriers to cancer screenings for people with ID (and where autistic people were included within some of these studies) found eight common barriers including a perception that screenings are distressing; previous negative interactions with HCP; a sense of being singled out, embarrassed, or unprepared; a lack of knowledge of cancer screening and procedural details; mobility issues; a high severity of ID; a lack of capacity to provide consent; and a lack of ability to communicate verbally (Chan et al., 2022). Of particular salience for cervical screening was the role of communication, from invitation through to follow-up correspondence (Levi et al., 2006; McIlfatrick et al., 2011).

Pregnancy and birth are other times when vaginal examinations, including using a speculum, transvaginal ultrasound, and a HCP’s fingers, occur. Literature considering autistic people’s experiences of maternity care highlights pertinent aspects relevant to sensory experiences, including difficulty being touched during physical examinations (Gardner et al., 2016), alongside poor communication by health professionals and inadequate analgesia when undertaking vaginal examinations (Grant et al., 2025). In addition, the clinical environment can represent a further sensory challenge, leading to increased difficulties communicating effectively with HCPs and “shutting down” (Donovan, 2020). As cervical screening examinations require both vaginal examination and attendance at a medical setting, it is plausible that these challenges remain salient.

According to the Theory of Planned Behaviour (TPB, Ajzen, 1985), three factors influence health-related behaviour: (1) individual intentions to perform the required behaviour (i.e., attend a screening), (2) beliefs about what other significant people will endorse (i.e., other people believe attendance is important), and (3) the extent to which the individual can control their behaviour (i.e., they are able to attend an appointment). These elements have been demonstrated for their relevance in predicting general attendance at cervical (Roncancio et al., 2015), colorectal (Huang et al., 2020), and breast cancer (Drossaert et al., 2003) screening uptake.

To the best of our knowledge, there is no current literature that examines the experience of cervical screening examinations with a focus on autistic people. This study therefore aimed to explore autistic people’s experience of, and attitudes towards, cervical screening to specifically explore the following:

1. The role of attitudes, perceived behavioural control, intention to attend, social norms, and knowledge about cervical screening between those who have and have not attended a cervical screening appointment.
2. Whether perceptions of pain are implicated in those who have and have not attended a cervical screening appointment.
3. Whether there will be a difference in sensory and communication issues between those who have and have not attended a cervical screening appointment.

## Method

### Design

An online questionnaire mixed-methods cross-sectional design was used. Participants were asked to provide responses to both standardised psychometric measures as well as study-specific open and closed questions to give additional depth of understanding.

### Participatory Methods

An academic autistic co-supervisor (B.D.) was involved throughout all stages of study development including raising the initial conception for the research, involvement in the design of the research, and building the measures and completion of the write-up to help ensure study accessibility and inclusivity. B.D. was an autistic PhD student under the supervision of D.M. at the time of the research. An autistic academic with expertise in reproductive healthcare for autistic people (A.G.) was involved in interpretation and writing up the study.

### Participants

Participants (*N* = 97) consisted of autistic (formal or self-diagnosed) people with a cervix. Self-diagnosis was included, as it is understood that autism is underdiagnosed in people with a cervix (Jamison et al., 2017). Participants were aged between 24 and 65, corresponding to the age range of those invited for regular cervical screenings in the United Kingdom (National Health Service [NHS], 2020, March 3rd). Participants included both those who had never or had attended for a cervical screening. All participants were able to provide consent for their own participation and reported independent living.

### Measures

#### Demographic Characteristics

Age, gender identity, ethnicity, and occupation were recorded. Participants were asked whether they considered themselves to have experienced sexual assault (yes, no, prefer not to say). This was recorded due to an identified association between cervical screening (or absence thereof) and historic sexual abuse (Hindin et al., 2019).

#### Cervical Screening Attendance

A history of ever having attended a cervical screening (yes/no) and a proximal estimate of the time since attending a screening (months, years) were recorded. If participants were unsure, they were asked to give their best estimate. Using this latter information, two categories were created to denote attendance “on time” (within the timeframe, according to age, as specified by NHS England), and “delayed.”^1^

#### Attitudes Towards Cervical Screening

Attitudes towards cervical screening appointments were measured using the TPB (Ajzen, 1985) and its four constructs: Intention, Attitudes, Subjective Norms, and Perceived Behavioural Control. One question assessed intention to attend next screening; “Do you plan on attended your next scheduled cervical screening examination?”, to which participants could answer “yes” or “no”. Nine statements followed, with three items per three remaining constructs: attitudes, perceived behavioural control, and subjective norms. Participants were asked to indicate their agreeableness to these statements on a five-point Likert-type scale (1 = *strongly agree*, 2 = *agree*, 3 = *neither agree nor disagree*, 4 = *disagree*, and 5 = *strongly disagree*). The measures were adapted from an already published study aiming to understand TPB in attendance at cervical screenings (Roncancio et al., 2015). An example item includes, “my closest friends would want me to have a cervical screening examination” (subjective norm). All subscales demonstrated high internal consistency for the present study: attitudes towards screening (α = .70), subjective norms (α = .84), and perceived behavioural control (α = .92).

#### Pelvic Pain

Pelvic pain was measured using nine statements from the British Society for Gynecological Endoscopy (BSGE) Pelvic Pain Questionnaire (Byrne et al., 2018). Participants were asked to rate the severity of nine symptoms associated with pre-menstrual pain, menstrual pain, non-cyclical pelvic pain, pain during sexual intercourse, pain opening bowels during period and at other times, bladder pain or pain passing urine, difficulty emptying the bladder, and lower back pain. Items were scored on a scale of 1 to 10. Internal consistency of this measurement tool for the present study was excellent (α = .87).

#### Pain Catastrophising

Pain catastrophising was measured with the short-form Pain Catastrophising Scale (PCS; Sullivan et al., 1995), which records rumination, helplessness, and magnification. The measure includes 13 statements, scored on a 5-point scale (0–4). An example item from this measure is “I worry all the time about whether the pain will end”. The PCS total score (α = .96) and all three subscales, rumination (α = .94), magnification (α = .86), and helplessness (α = .92), all demonstrated high internal consistency when calculated from the present study.

#### Fear of Pain

Fear of pain was measured with the short-form Fear of Pain Questionnaire-9 (FPQ; McNeil et al., 2018). Nine statements are scored on a scale of 1–4. An example item from this measure includes “I fear the pain associated with getting a papercut on your finger.” The FPQ total score (α = .83) and all three subscales, severe (α = .75), minor (α = .72), and medical (α = .79), all demonstrated high internal consistency calculated from this study.

#### Experience of Healthcare Provider Communication

Items were adapted from Raymaker et al.’s (2017) “Barriers to Healthcare Checklist,” which had been developed to be used with autistic people. Communication between the person accessing the screening, the healthcare system, and the individual conducting the screening was measured using nine items scored on a scale of 1–5. All participants were shown these questions regardless of previous attendance and had the option to leave the answer blank if not applicable. An example statement for this measure was “The information provided to me about my appointment, and any materials about the procedure, was easy to understand”. Internal consistency for this measure (α = .66) was questionable for the present study.

#### Sensory Issues

Items to measure sensory issues were also adapted from Raymaker et al. (2017) to capture sensory issues pertaining to (1) an overall experience of accessing healthcare and (2) sensory issues specific to the cervical screening appointment. Four statements were included, scored from 1 to 5. An example question includes “Sensory Issues (e.g., sensitivity to lights, sounds or smells) make screenings and medical examinations, such as a cervical screening difficult or impossible”. Internal consistency for this measure (α = .76) was acceptable as calculated from the present study.

#### Knowledge of Cervical Screening

Knowledge about the U.K. cervical screening and vaccination programme was measured using two items from the “Cervical Cancer Awareness Measure” (Cervical CAM; Simon et al., 2012), scored as yes or no. An example item from this measure includes “As far as you are aware, is there an NHS vaccine available to protect against cervical cancer?” Participants are asked to answer with yes or no. An affirmative answer prompts a further question enquiring about the age of screening in the United Kingdom. Internal consistency was poor for this measure as calculated from the present study (α = .23).

#### Overall Experience

A series of open-ended questions was included, tailored for participants who had/had not attended a cervical screening. Participants who had attended a screening were provided with the following: “We are going to ask you to describe your experience of a cervical screening. Below are some examples of what you might want to discuss as part of the ‘experience’.” Participants were also asked, “Were there any factors or arrangements which made attending your last cervical screening appointment easier?” and “Was there anything which was not offered which you would like to be offered in future” with free-text response boxes. Out of those who had previously attended a screening, 51 provided (of 82 eligible) responses to the free-text questions.

Participants who had not attended a screening instead were provided with the following: “Please describe in as much detail as you feel comfortable your main reasons for not attending a cervical screening appointment” with a free-text response box. They were also asked, “Is there anything which could be offered or considered which would make you more likely to attend a cervical screening?” Out of those who had never previously attended, 12 of 15 provided responses to the free-text questions.

### Procedures

Ethical approval was obtained from the School of Psychology Research Ethics Committee at Liverpool John Moores University. A poster containing study information and link to the participant information sheet and online questionnaire, hosted by Qualtrics, was distributed via social media outlets. Specific social media groups aimed at supporting autistic people were also approached. There was no compensation or incentive to take part.

### Data Analysis

Descriptive statistics were computed for demographic variables. Normality tests were conducted, and bivariate tests of difference (independent *t*-tests or Mann–Whitney *U*) were computed with study variables and cervical screening attendance. Where bivariate significance was identified, logistic regression was conducted.

Qualitative data were analysed using Thematic Analysis (Clarke et al., 2015). Thematic Analysis consists of six stages: data familiarisation, coding, searching for themes, reviewing themes and patterns, defining themes, and producing the report (Clarke et al., 2015). First, data were coded by attendance group and then grouped together when beginning to generate themes across the data. The data encompassed their experiences of screening (if previously attended), the barriers to attendance, and any facilitators to attendance or recommendations for future practice. Robustness was ensured throughout the analysis by checking and re-checking against the data sets and discussing with supervisors.

### Results

#### Participant Characteristics

Participants were aged on average 36.15 years (*SD* = 11.09, range = 24–61). The majority of participants were female (*N* = 78, 81%), white (*N* = 90, 95%), currently in full-time employment (*N* = 43, 44%), had completed an undergraduate degree (*N* = 44, 45%), and were formally diagnosed as autistic (*N* = 53, 55%) See table 1 for more details. The high number of participants in this study likely reflects the general later diagnosis of autistic people assigned female at birth (Barabasz et al., 2025); this is also a population who might have unique healthcare experiences (Gillions et al., 2026). Most of the participants had attended at least one cervical screening (*N* = 82, 85%). To facilitate exploration into the relevancy of factors that may explain attendance at a cervical screening, a proxy variable was computed to distinguish participants who (1) had attended a cervical screening appointment as required (*N* = 65) and (2) who had not and/or who were delayed in accessing their screening appointment (*N* = 32).

**Table 1.** Sample Characteristics and Demographic Data Including Diagnostic Status, Gender Identity, and Previous Engagement in Cervical Screening.

Variable	*N*	%^a^
Autism diagnosis		
Formal diagnosis	53	55%
No formal diagnosis/on diagnosis pathway	19	19%
No formal diagnosis/not on diagnosis pathway	23	24%
No response	2	2%
Previous attendance at screening		
Yes	82	85%
No	15	15%
Gender identity		
Female	78	81%
Non-binary	12	12%
Transgender	2	2%
Male	1	1%
Prefer not to say	1	1%
Prefer to self-describe	1	1%
No response	2	2%
Ethnicity		
White	90	95%
Mixed multiple background	2	2%
White and Asian	1	1%
Indian	1	1%
Any other Asian background	1	1%
No response	2	2%
Employment status		
Full-time employment	43	44%
Part-time employment	18	19%
Unemployed	14	15%
Student	8	8%
Retired	5	5%
Prefer not to say	6	5%
No response	3	3%
Level of education		
Undergraduate	44	45%
Postgraduate	30	31%
Higher education (GCSE/A-level)	18	19%
Prefer not to say	2	2%
No response	3	3%

*N* = 97.

a % denotes the proportion of all participants.

#### Examining Group Differences

There was no significant difference between groups (attended vs. not attended/delayed) for the following variables: sensory issues, communication difficulties, fear of pain (FPQ) total score and subscales, pain catastrophising (total score and subscales), knowledge of cervical screening, or experience of sexual assault (*ps* > .05)

There was a significant difference found for intention to attend the next screening between those who had attended and those with delayed/no attendance, χ^2^(1, 97) = 11.85, *p* = .002. Those with previous attendance experience are more likely to express an intention to attend (*N* = 67, 81.7% of those with previous attendance), in comparison to those with delayed/no attendance (*N* = 6, 40% of those with delayed/no attendance).

Following a similar pattern, there were significant differences between those who had/had not or delayed screening attendance for attitudes towards screening and perceived behavioural control. Those with previous on-time screening attendance reported more favourable attitudes towards screening (*U* = 402.00, *z* = −2.13, *p* = .033) and a higher level of perceived behavioural control (*U* = 847.50, *z* = 2.33, *p* = .020). There was no significant difference between groups based on subjective norms (*U* = 688.50, *z* = .74, *p* = .460). Further details are displayed in Table 2.

**Table 2.** Differences in Theory of Planned Behaviour Facets (TPB) by Screening Attendance Groups (Attended vs. Non-Attended or Delayed Attendance).

TPB facet	Median (range)	
	Attended (*N* = 82)	Delayed/no attendance (*N* = 15)
Subjective norms	12.54 (3.23-15.34)	12.20 (8.56-15.00)^a^
Perceived behavioural control	9.57 (3.00-15.00)	8.00 (3.00-13.00)*
Attitudes towards screening	10.40 (3.00-15.00)	12.00 (9.00-14.00)*

*N* = 97, *p *<* .05, ^a^*p* = .460.

#### Factors Associated With Screening Attendance

Binary logistic regression was used to examine whether intention to attend the next screening, perceived behavioural control, subjective norms, and attitudes towards cervical screening (Independent Variables: IVs) were associated with attendance at cervical screening (Dependent Variable: DV). The model was statistically significant, χ^2^ (2, 97) = 27.48, *p* < .001. The model explained between 24.7% (Cox & Snell *R*^2^) and 34.3% (Nagelkerke *R*^2^) of the variance in the dependent variable and correctly classified 79.4% of cases. As shown in Table 3, intention to attend the next screening was the only variable to contribute to the model independently and significantly. This suggests that those who have no intention to attend their next cervical screening will have a less likely chance of attending. It also highlights the limited role of subjective norms and perceived behavioural control in understanding screening attendance behaviours.

**Table 3.** Logistic Regression Predicting Historic Attendance at Cervical Screening From Theory of Planned Behaviour Variables.

							95% CI	
Predictor	*B*	*SE*	Wald	*df*	*p*	*OR*	*LL*	*UL*
Subjective norms	0.14	0.12	1.57	1	.210	1.15	0.92	1.43
Perceived behavioural control	-0.17	0.11	0.02	1	.880	0.98	0.79	1.22
Attitudes towards screening	-.07	0.12	0.37	1	.546	0.93	0.73	1.17
Intention to attend screening	-2.57	0.78	10.84	1	.001	0.08	0.02	0.35
Constant	3.33	2.42	1.89	1	.169	0.17		

*N* = 97.

#### Exploring Perspectives on Screening Attendance

A total of 66 participants provided written responses to at least one open-ended question (*N* = 53 who had attended screening, *N* = 13 who had not or who had delayed attendance). Two over-arching themes of “Communication disconnect across the care journey” and “Echoes of the past: the lasting impact of previous care encounters” were generated, with subthemes of communication pre-appointment, communication with a healthcare provider, negative previous experiences, and anticipating a negative experience. Themes and subthemes are described below alongside illustrative quotations.

### Theme 1: Communication Disconnect Across the Care Journey

This theme encompasses perspectives about the quality and appropriateness of communication received prior to and during the screening appointment. It highlights communication-related barriers to screening uptake throughout the patient journey, which is initiated by the appointment letter, and that communication from this point onwards is of importance. Within this theme, there were two subthemes reflecting views regarding communication prior to the appointment and during.

#### Pre-Appointment Matters: Gaps in Pre-Appointment Communication

Accessibility, particularly regarding communication, was a source of both frustration and distress. Issues with communication were found at different stages in the screening process, starting with the invitation to attend:

The letter was too long for me and without visuals so I had to ask my mum for information about the experience. (Ppt 32)

Furthermore, the process of booking the appointment was an additional source of anxiety:

Getting the appointment organised was very stressful and confusing . . . . As I did not understand what procedure I was going to have and what was going to happen. (Ppt 21)

This is suggestive of similar issues with the communication style of the invite. Others also found the mode of booking an appointment, largely via telephone, to be anxiety-provoking. In one case, this resulted in a participant delaying attending their screening:

I find it very difficult to make appointments and hate talking on the phone. I had my screening years after the first invitation. (Ppt 42)

#### Misaligned Communication With Healthcare Providers

Participants spoke of further issues with communication and the interaction between them and the professional conducting the screening:

I felt like the questions at the beginning of the appointment were random – she asked me about my period and didn’t really explain why she needed to know this . . . I felt like I couldn’t ask WHY!!! I like clear answers to things. (Ppt 22)

This emphasises a lack of clarity in communication and the functional aspects of screening indicative of limited understanding from healthcare practitioners around the importance of communication or the communication needs of autistic people.

Positive interactions with HCPs were noted by some and made clear that the nature of the interaction had the power to change the tone of the appointment. For example, the participant below emphasised the importance of feeling listened to in the context of being autistic and how this transformed a previously traumatic experience into a positive one:

The last screening was a breeze because the nurse *listened*, I’d explained that I’m autistic . . . She took all of that on board and the procedure was very straightforward, the previous ones had all been traumatic. (Ppt 73)

### Theme 2: Echoes of the Past: The Lasting Impact of Previous Care Encounters

This theme emphasises the role of previous experience in shaping anticipation and expectations of participants with regard to future screening appointments. Previous experience encompassed specific screening appointments but also observations from interactions with healthcare providers more widely. For those without screening attendance, the focus tended to be on anticipated challenges in managing responses during an appointment. Similarly, previous experience shaped expectations.

#### Expecting the Worst: The Lasting Impact of Negative Encounters

Many participants commented on previous healthcare experiences and the lasting impact they have had. One participant named previously felt “dismissed” as a large contributory factor to their decision to not attend a cervical screening examination:

I’ve had bad experiences with GPs when communicating about my problems. They weren’t patient with me and dismissed my concerns. (Ppt 6)

For those who have attended, multiple disclosed traumatic experiences have led to a delay in attending for their cervical screening:

I tried to explain I was nervous . . . . He then really hurt me with the speculum and I lashed out with my foot and he abandoned the procedure. It’s taken 6 years to psyche myself up for a smear test. (Ppt 76)

Another participant describes, a similar experience, in not being listened to when trying to communicate distress and with traumatic consequences. This participant reported feeling as if HCPs are not empathetic around the difficulty of attending a screening:

I find screening very difficult due to sensory issues and my past sexual abuse. I feel that they don’t really understand or have knowledge of how screening can be so hard, they just assume it’s embarrassment on my part rather than trauma, etc. (Ppt 83)

Others also spoke about the overwhelming nature of the appointment, with some reporting traumatic consequences:

Everyone was kind, but the experience was so painful and traumatising that I was screaming and crying and we had to stop. Couldn’t even get the speculum in. (Ppt, 46)

#### “Not Just the Appointment”: Anticipatory Concern Over Coping

For those who have never attended, many were anticipating an inability to cope with the situation within the remits of the appointment:

I’m afraid of having a panic attack or shutdown, and not having enough time to recover during the appointment. I’ve also found that in the past that having a panic attack can make it difficult for me to leave the home for months afterwards. As a result I would do almost anything to avoid having another one. (Ppt 9)

This excerpt highlights multiple distressing factors. The participant anticipates anxiety during the screening itself, without suitable accommodations to manage this; in this instance, the length of the appointment. They also discuss the potential long-term anxiety attached to attending, which they do not perceive to be worth the risk. It appears that others also anticipate similarly high levels of distress when considering attending cervical screening:

Would rather die of cancer. (Ppt 2)

## Discussion

This study aimed to provide an exploratory insight into autistic people’s experiences of and attitudes towards cervical screenings. It also aimed to look at already established barriers to screening and understand how these may impact autistic people’s decision to attend a cervical screening and explore any additional barriers which may be more pertinent to this group, including communication and sensory difficulties. Suggestions to improve the experience of a cervical screening for autistic people were also considered.

Attitudes towards screening (measured using TPB; Ajzen, 1985) were a relevant factor in understanding of screening attendance, particularly a person’s intention to attend their next screening. TPB is already well established in predicting screening behaviour within non-autistic populations (Drossaert et al., 2003; Huang et al., 2020; Roncancio et al., 2015), with our findings suggesting that it is also able to predict screening behaviour within an autistic sample. This reinforces the potential practical application of the TPB for this population. Elements of the TPB are already used as intervention targets to improve uptake within the general population; an example of this is the most recent cervical screening campaign, “Help us help you” running in the United Kingdom (Department of Health & Social Care, 2022). With the knowledge that TPB can predict autistic people’s screening behaviour, there are promising implications for the use of such interventions with this population too.

Quantitatively, none of the measures for pain or communication difficulties differed by attendance group, but they did feature in the qualitative data. Both the FPQ and PCS are validated measures which are regularly used in pain research due to their high validity and reliability (McNeil et al., 2018; Osman et al., 1997); such measures have also been used with autistic participants in pain research, yielding some significant results (Failla et al., 2020). Feedback within the qualitative data stated that some participants would have liked a “not sure” or “don’t know” option for items on the questionnaire, suggesting that these participants may not have understood the questions being asked or the relevance within the context of a study about cervical screenings.

A key clinical implication of this study is the importance of HCP interaction. For a minority, this was the difference in turning a previously traumatic experience into a manageable and safe experience. Satisfaction with HCP’s attitudes has been found to be a significant mitigator of post-traumatic stress following childbirth (Çapik & Durmaz, 2018). While cervical screening is different in nature to childbirth, it is similarly invasive, and therefore, it could be suggested that a positive healthcare interaction could similarly mitigate post-screening trauma. It is clear therefore that more work to support HCPs in understanding and engaging with autistic patients in all specialisms will benefit clinical engagement and outcomes for this group. These improvements might involve a multifaceted strategy including training of staff within specialised services to support these patients’ needs; this might include general training to upskill all staff but also specialist training for staff to support patients with additional needs, as well as longer appointment durations and trauma informed care.

Participants highlighted that uncertainty and not knowing what to expect were a large contributor to anxiety and distress. A feasible suggestion could be to utilise technology to prepare patients for the procedure. One participant suggested providing a virtual tour of the venue or screening procedure before the appointment could ease anxiety. Although accessible guides to cervical screening are available, NHS England recommends the use of these guides at the discretion of general practitioner (GP) practices. This often relies on patients being formally being diagnosed and documented as autistic, a well-known issue for people with a cervix (Jamison et al., 2017). NHS England (2020, March 30th) also suggests that pre-appointments should be offered as an opportunity to ask questions about the procedure or discuss previous negative experiences. The findings from this research suggest that this guidance is relevant yet requires further uptake and/or reinforcement. Furthermore, it might be beneficial for these opportunities to be particularly highlighted where a patient might be more likely to be vulnerable in these clinical encounters. These processes could form an aspect of universal design to ensure all who might benefit from these accommodations can utilise them and could be included with all invitation letters through QR codes.

It is further clear here that the development of better cervical screening experiences for autistic people needs to fit within a wider strategy to improving overall experiences of healthcare for this population. In our study, autistic people reported high trauma experiences during screening and the potential role that a history of sexual abuse might play here, which is unfortunately more common (Trundle et al., 2023). Furthermore, autistic-specific issues such as a fear of either meltdown or shutdown might exacerbate concerns about engaging with treatments. This potentially plays into the expectation of poorer quality healthcare that we found here and in previous studies (Doherty et al., 2022). By improving care and engaging better with autistic patients, it is possible that these expectations can be reversed and that engagement with healthcare will improve and outcomes also. It is critical the individual patient needs are considered here; in particular, a lack of consideration of sensory sensitivities are not systematically made within appointments. In particular, here, one participant reported that they informed HCPs about their fears and that they continued and caused pain, resulting in discontinuation of the procedure. Here, it is evident that a clear approach to listen to and respond to the concerns of patients, in particular in this case, showing an awareness of autistic patient needs.

As an entirely exploratory investigation, this research provides promising insight into the factors that are implicated in autistic people’s attendance for cervical screening. Findings are limited by the small sample size and relatively small proportion of individuals who had never attended a screening. The diversity of the participant pool, predominantly White and highly educated, also requires acknowledgement with recommendations for future research to incorporate further efforts to involve underserved parts of the autistic community. In particular, individuals with co-occurring IDs might have specific/additional barriers and needs in accessing these services. This limitation is particularly important as the experiences of autistic people of non-White identities are particularly underrepresented (Hilton et al., 2010). It is further relevant here that the present research aimed to only consider the experience of an autistic population; it is likely that some of the themes identified in the current research likely also apply to non-autistic people when engaging in cervical screening. This research was not intended to identify uniquely autistic experiences but rather to start a narrative around autistic experiences of cervical screening and provide a forum for these seldom-heard voices. The aim here is to provide a framework for future research to consider how barriers to engagement might be reduced for autistic people. It is recognised that the creation of a binary “attended” versus “not attended/delayed” variable has its limitations, primarily due to its simplicity, but it was deemed appropriate for this small exploratory study and served as a useful foundation upon which to initiate comparison that may explain this health-related behaviour. In the context of the current research, this might be especially important as cultural differences might also alter engagement with cervical screen procedures (Marlow et al., 2015). Given that individually these factors might affect engagement, it is important to understand the intersectional relationship between autism and ethnicity and its effect on cervical screen attendance. The survey was carefully designed following a scoping review of relevant literature and measures selected on this basis. It must be noted that the acceptability and appropriateness of questionnaires were in places limited for autistic participants, highlighting recommendations for future research to specifically examine and potentially develop specific measurement tools for this population.

This research highlights the need to improve healthcare communication and other accessibility needs for autistic people when attending cervical screening or other healthcare appointments. Such interventions should be co-developed by autistic people. When surveyed, only the intention to attend a screening explained differences between individuals who had attended a screening and those who had not/were delayed in their uptake, although this may have been due to sample size. Difficulties with communication and accessibility were prominent through qualitative feedback. Further in-depth investigation is required to increase understanding from both the perspective of autistic people and HCPs, to ultimately enhance service provision within the context of cervical screening.

## Notes

1. This corresponded to a screening taking place over 36 months for participants aged 24–29 years and over 5 years for participants aged 50–65 years.

## References

[bibr1-13623613261439937] Ajzen I. (1985). From intentions to actions: A theory of planned behavior. In Kuhl J. Beckmann J. (Eds.), Action control (pp. 11–39). Springer.

[bibr2-13623613261439937] Barabasz K. M. Baran J. Barczuk P. Bańkowski D. Bielak K. Kamińska K. Kłaptocz P. Łukoś-Karcz K. Nowak M. (2025). Underdiagnosis of autism spectrum disorder: A narrative review of gender differences and systemic barriers. Advances in Autism, 12, 136–146.

[bibr3-13623613261439937] Boshoff K. Bowen-Salter H. Gibbs D. Phillips R. L. Porter L. Wiles L. (2021). A meta-synthesis of how parents of children with autism describe their experience of accessing and using routine healthcare services for their children. Health & Social Care in the Community, 29(6), 1668–1682. 10.1111/hsc.13369 34155717

[bibr4-13623613261439937] Byrne D. Curnow T. Smith P. Cutner A. Saridogan E. Clark T. J. (2018). Laparoscopic excision of deep rectovaginal endometriosis in BSGE endometriosis centres: A multicentre prospective cohort study. BMJ Open, 8(4), Article e018924. 10.1136/bmjopen-2017-018924 PMC5892761 29632080

[bibr5-13623613261439937] Cancer Research UK. (2015). Cervical cancer statistics. https://www.cancerresearchuk.org/healthprofessional/cancer-statistics/statistics-by-cancer-type/cervical-cancer#heading-Five

[bibr6-13623613261439937] Çapik A. Durmaz H. (2018). Fear of childbirth, postpartum depression, and birth-related variables as predictors of posttraumatic stress disorder after childbirth. Worldviews on Evidence-Based Nursing, 15(6), 455–463. 10.1111/wvn.12326 30281197

[bibr7-13623613261439937] Chan D. N. Law B. M. Au D. W. So W. K. Fan N . (2022). A systematic review of the barriers and facilitators influencing the cancer screening behaviour among people with intellectual disabilities. Cancer Epidemiology, 76, 102084. 10.1016/j.canep.2021.102084 34920342

[bibr8-13623613261439937] Chen Y. Powers J. McDougle C. J. Zürcher N. R. Thom R. P. (2024). Cervical cancer screening and prevention uptake in females with autism spectrum disorder. Journal of Autism and Developmental Disorders, 56, 1–12. 39294385 10.1007/s10803-024-06565-2

[bibr9-13623613261439937] Chorley A. J. Marlow L. A. Forster A. S. Haddrell J. B. Waller J. (2017). Experiences of cervical screening and barriers to participation in the context of an organised programme: A systematic review and thematic synthesis. Psycho-Oncology, 26(2), 161–172. 10.1002/pon.4126 27072589 PMC5324630

[bibr10-13623613261439937] Clarke V. Braun V. Hayfield N. (2015). Thematic analysis. Qualitative Psychology: A Practical Guide to Research Methods, 222(2015), 248.

[bibr11-13623613261439937] Corden K. Brewer R. Cage E. (2021). A systematic review of healthcare professionals’ knowledge, self-efficacy and attitudes towards working with autistic people. Review Journal of Autism and Developmental Disorders, 9, 386–399. 10.1007/S40489-021-00263-w

[bibr12-13623613261439937] Crane L. Adams F. Harper G. Welch J. Pellicano E . (2019). ‘Something needs to change’: Mental health experiences of young autistic adults in England. Autism, 23(2), 477–493. 10.1177/1362361318757048 29415558

[bibr13-13623613261439937] Department of Health & Social Care. (2022, February 14). New national cervical screening campaign launches – as nearly 1 in 3 don’t take up screening offer. https://www.gov.uk/government/news/new-national-cervical-screening-campaign-launches-as-nearly-1-in-3-dont-take-up-screening-offer

[bibr14-13623613261439937] Doherty M. Neilson S. O’Sullivan J. Carravallah L. Johnson M. Cullen W. Shaw S. C. (2022). Barriers to healthcare and self-reported adverse outcomes for autistic adults: A cross-sectional study. BMJ Open, 12(2), Article e056904. 10.1136/bmjopen-2021-056904 PMC8883251 35193921

[bibr15-13623613261439937] Donovan J. (2020). Childbirth experiences of women with autism spectrum disorder in an acute care setting. Nursing for Women’s Health, 24(3), 165–174. 10.1016/j.nwh.2020.04.001 32389581

[bibr16-13623613261439937] Douglas E. Waller J. Duffy S. W. Wardle J . (2016). Socioeconomic inequalities in breast and cervical screening coverage in England: Are we closing the gap? Journal of Medical Screening, 23(2), 98–103. 10.1177/0969141315600192 PMC4855247 26377810

[bibr17-13623613261439937] Drossaert C. H. C. Boer H. Seydel E. R. (2003). Prospective study on the determinants of repeat attendance and attendance patterns in breast cancer screening using the theory of planned behaviour. Psychology & Health, 18(5), 551–565. 10.1080/0887044031000141207

[bibr18-13623613261439937] Failla M. D. Gerdes M. B. Williams Z. J. Moore D. J. Cascio C. J. (2020). Increased pain sensitivity and pain-related anxiety in individuals with autism. Pain Reports, 5(6), Article e861. 10.1097/PR9.0000000000000861 PMC7676593 33235944

[bibr19-13623613261439937] Gardner M. Suplee P. D. Bloch J. Lecks K. (2016). Exploratory study of childbearing experiences of women with Asperger syndrome. Nursing for Women’s Health, 20(1), 28–37. 10.1016/j.nwh.2015.12.001 26902438

[bibr20-13623613261439937] Gillions A. O’Nions E. Mansour H. Hoare S. Mandy W. Stott J. (2026). The healthcare experiences of middle and older age autistic women in the United Kingdom. Autism, 30(1), 49–60. 40792546 10.1177/13623613251362265 PMC12717282

[bibr21-13623613261439937] Grant A. Griffiths C. Williams K. Brown A . (2025). “I just gritted my teeth to get through it all”: An online survey of autistic people’s experiences of maternity care in the United Kingdom. Autism in Adulthood. Advance online publication. 10.1089/aut.2024.0275

[bibr22-13623613261439937] Hilton C. L. Fitzgerald R. T. Jackson K. M. Maxim R. A. Bosworth C. C. Shattuck P. T. Geschwind D. H. Constantino J. N. (2010). Brief report: Under-representation of African Americans in autism genetic research: A rationale for inclusion of subjects representing diverse family structures. Journal of Autism and Developmental Disorders, 40, 633–639. 19936905 10.1007/s10803-009-0905-2 PMC3645854

[bibr23-13623613261439937] Hindin P. Btoush R. Carmody D. P. (2019). History of childhood abuse and risk for cervical cancer among women in low-income areas. Journal of Women’s Health, 28(1), 23–29. 10.1089/jwh.2018.6926 30265615

[bibr24-13623613261439937] Hu K. Wang J. Sparén P. Herweijer E. Sjölander A. Adami H. O. Valdimarsdóttir U. Sundström K. Fang F. (2023). Invasive cervical cancer, precancerous lesions, and cervical screening participation among women with mental illness in Sweden: A population-based observational study. The Lancet Public Health, 8(4), e266–e275. 10.1016/S2468-2667(23)00026-9 36965981

[bibr25-13623613261439937] Huang J. Wang J. Pang T. W. Chan M. K. Leung S. Chen X. Leung C. Zheng Z. J. Wong M. C. (2020). Does theory of planned behaviour play a role in predicting uptake of colorectal cancer screening? A cross-sectional study in Hong Kong. BMJ Open, 10(8), Article e037619. 10.1136/bmjopen-2020-037619 PMC7412617 32764087

[bibr26-13623613261439937] Jamison R. Bishop S. L. Huerta M. Halladay A. K. (2017). The clinician perspective on sex differences in autism spectrum disorders. Autism: The International Journal of Research and Practice, 21(6), 772–784. 10.1177/1362361316681481 28429618

[bibr27-13623613261439937] Levi M. Kimpton B. Sim J. (2006). Women with learning disabilities’ and carers’ experiences and views of cervical screening: A research project undertaken in South Central and Southwest Edinburgh LHPs and in Lothian. https://www.choiceforum.org/docs/hwpfinal.pdf

[bibr28-13623613261439937] Lum M. Garnett M. O’Connor E. (2014). Health communication: A pilot study comparing perceptions of women with and without high functioning autism spectrum disorder. Research in Autism Spectrum Disorders, 8(12), 1713–1721. 10.1016/j.rasd.2014.09.009

[bibr29-13623613261439937] Marlow L. Chorley A. J. Rockliffe L. Waller J. (2018). Decision-making about cervical screening in a heterogeneous sample of nonparticipants: A qualitative interview study. Psycho-Oncology, 27(10), 2488–2493. 10.1002/pon.4857 30095862 PMC6220875

[bibr30-13623613261439937] Marlow L. A. Waller J. Wardle J. (2015). Barriers to cervical cancer screening among ethnic minority women: A qualitative study. Journal of Family Planning and Reproductive Health Care, 41(4), 248–254. http://dx.doi.org/10.1136/jfprhc-2014-101082 25583124 10.1136/jfprhc-2014-101082 PMC4621371

[bibr31-13623613261439937] McIlfatrick S. Taggart L. Truesdale-Kennedy M. (2011). Supporting women with intellectual disabilities to access breast cancer screening: A healthcare professional perspective. European Journal of Cancer Care, 20(3), 412–420. 10.1111/j.1365-2354.2010.01221.x 20825462

[bibr32-13623613261439937] McNeil D. W. Kennedy S. G. Randall C. L. Addicks S. H. Wright C. D. Hursey K. G. Vaglienti R. (2018). Fear of pain questionnaire-9: Brief assessment of pain-related fear and anxiety. European Journal of Pain (London, England), 22(1), 39–48. 10.1002/ejp.1074 28758306 PMC5730485

[bibr33-13623613261439937] Morris R. Greenblatt A. Saini M . (2019). Healthcare providers’ experiences with autism: A scoping review. Journal of Autism and Developmental Disorders, 49(6), 2374–2388. 10.1007/s10803-019-03912-6 30758692

[bibr34-13623613261439937] National Health Service. (2020, March 3). Cervical screening: Ideas for improving access and uptake. https://www.gov.uk/guidance/cervical-screening-ideas-for-improving-access-and-uptake#women-with-specific-needs-or-with-disabilities

[bibr35-13623613261439937] National Health Service. (2020, March 30). Cervical screening – why is it important? https://www.nhs.uk/conditions/cervical-screening/why-its-important/

[bibr36-13623613261439937] Nicolaidis C. Raymaker D. McDonald K. Dern S. Boisclair W. C. Ashkenazy E. Baggs A. (2013). Comparison of healthcare experiences in autistic and non-autistic adults: A cross-sectional online survey facilitated by an academic-community partnership. Journal of General Internal Medicine, 28(6), 761–769. 10.1007/s11606-012-2262-7 23179969 PMC3663938

[bibr37-13623613261439937] Osman A. Barrios F. X. Kopper B. A. Hauptmann W. Jones J. O’Neill E. (1997). Factor structure, reliability, and validity of the Pain Catastrophizing Scale. Journal of Behavioral Medicine, 20(6), 589–605. 10.1023/A:1025570508954fear 9429990

[bibr38-13623613261439937] Raymaker D. M. McDonald K. E. Ashkenazy E. Gerrity M. Baggs A. M. Kripke C. Hourston S. Nicolaidis C. (2017). Barriers to healthcare: Instrument development and comparison between autistic adults and adults with and without other disabilities. Autism: The International Journal of Research and Practice, 21(8), 972–984. 10.1177/1362361316661261 27663266 PMC5362353

[bibr39-13623613261439937] Roncancio A. M. Ward K. K. Sanchez I. A. Cano M. A. Byrd T. L. Vernon S. W. Fernandez-Esquer M. E. Fernandez M. E. (2015). Using the theory of planned behavior to understand cervical cancer screening among Latinas. Health Education & Behavior: The Official Publication of the Society for Public Health Education, 42(5), 621–626. 10.1177/1090198115571364 25712240 PMC4932857

[bibr40-13623613261439937] Shin D. W. Lee J. W. Jung J. H. Han K. Kim S. Y. Choi K. S. Park J. H. Park J. H. (2018). Disparities in cervical cancer screening among women with disabilities: A national database study in South Korea. Journal of Clinical Oncology, 36(27), 2778–2786. 10.1200/JCO.2018.77.7912 30074846

[bibr41-13623613261439937] Simon A. E. Wardle J. Grimmett C. Power E. Corker E. Menon U. Matheson L. Waller J. (2012). Ovarian and cervical cancer awareness: Development of two validated measurement tools. The Journal of Family Planning and Reproductive Health Care, 38(3), 167–174. 10.1136/jfprhc-2011-100118 21933805 PMC3970720

[bibr42-13623613261439937] Sullivan M. J. L. Bishop S. R. Pivik J. (1995). The pain catastrophizing scale: Development and validation. Psychological Assessment, 7(4), 524–532. 10.1037/1040-3590.7.4.524

[bibr43-13623613261439937] Trundle G. Jones K. A. Ropar D. Egan V. (2023). Prevalence of victimisation in autistic individuals: A systematic review and meta-analysis. Trauma, Violence, & Abuse, 24(4), 2282–2296. 10.1177/15248380221093689 PMC10486169 35524162

[bibr44-13623613261439937] Wilding S. Wighton S. Halligan D. West R. Conner M. O’Connor D. B. (2020). What factors are most influential in increasing cervical cancer screening attendance? An online study of UK-based women. Health Psychology and Behavioral Medicine, 8(1), 314–328. 10.1080/21642850.2020.1798239 34040874 PMC8114340

